# The relationship between social networking addiction and academic performance in Iranian students of medical sciences: a cross-sectional study

**DOI:** 10.1186/s40359-019-0305-0

**Published:** 2019-05-03

**Authors:** Seyyed Mohsen Azizi, Ali Soroush, Alireza Khatony

**Affiliations:** 1 Clinical Research Development Center of Imam Reza Hospital, Kermanshah University of Medical Sciences, Kermanshah, Iran; 2 Social Development and Health Promotion Research Center, Kermanshah University of Medical Sciences, Kermanshah, Iran; 3 Nursing Department, School of Nursing and Midwifery, Dowlat Abad, Kermanshah, Iran

**Keywords:** Social networking, Addiction, Academic performance, University students, Bergen social media addiction scale

## Abstract

**Background:**

Social networks have had a major influence on students’ performance in recent years. These networks create many opportunities and threats for students in various fields. Addiction to social networking and its impact on students’ academic performance caused the researcher to design and conduct this study. The purpose of this study was to investigate the relationship between social networking addiction and academic performance of students in Iran.

****Method**s:**

In this cross-sectional study, 360 students were enrolled by stratified random sampling. The study tools included personal information form and the Bergen Social Media Addiction Scale. Also, the students’ overall grade obtained in previous educational term was considered as the indicator of academic performance. Data were analyzed using SPSS-18.0 and descriptive and inferential statistics.

**Findings:**

The mean social networking addiction was higher in male students (52.65 ± 11.50) than in female students (49.35 ± 13.96) and this difference was statistically significant (*P* < 0.01). There was a negative and significant relationship between students’ addiction to social networking and their academic performance (r = − 0.210, *p* < 0.01).

**Conclusions:**

The social networking addiction of the students was at moderate level and the male students had a higher level of addiction compared to the female students. There was a negative and significant relationship between the overall use of social networks and academic performance of students. Therefore, it is imperative that the university authorities take interventional steps to help students who are dependent on these networks and, through workshops, inform them about the negative consequences of addiction to social networks.

## Introduction

In recent years, significant changes have taken place around the world regarding the quantitative and qualitative expansion of internet, social networks and number of people who use them. Social networks include websites and applications that allow users to share content, ideas, opinions, beliefs, feelings, and personal, social, and educational experiences. They also allow communication between a wide range of users at global level [1, 2]. Instagram, Telegram, Facebook, Twitter, Skype, and WhatsApp are among the most popular and commonly used virtual social networks [3–8]. Currently (2018), the number of internet users in the world is about 4.021 billion and also 3.196 billion people use social networks on a regular basis worldwide [9]. Iran is one of the developing countries where internet and social networks have grown significantly. The use of social media has tripled over the past three years, and more than 47 million Iranians are using social networks, according to the Iranian Center of Statistics [10].

**Table 1:** Comparison of mean and standard deviation of social networking addiction score in terms of demographic characteristics

Variable		*n* (%)	Mean (SD)	*P*- value
Sex	Male	161 (44)	52.65 (11.50)	0.001
	Female	199 (55.3)	49.35 (13.96)	
Age group	≤20	19 (5.3)	53.78 (14.95)	
	21–30	310 (86.1)	51.54 (15.98)	NS^*^
	31–40	31 (8.6)	50.57 (11.45)	
Age (years), mean (SD)	25.48 ± 3.39	–	–	
Educational level	Undergraduate	168 (46.7)	52.8 (12.70)	
	Postgraduate	126 (35.0)	50.61 (12.75)	NS
	Ph.D.	66 (18.3)	48.03 (13.95)	
School	Medicine	41 (11.4)	51.73 (16.05)	
	Paramedical	95 (26.4)	53.49 (12.53)	
	Dentistry	101 (28.1)	50.43 (11.73)	NS
	Pharmacy	44 (12.2)	48.54 (12.54)	
	Nursing and Midwifery	50 (13.9)	48.08 (13.67)	
	Health	29 (8.9)	50.41 (12.84)	

^*^Non-significant

**Table 2:** Intensity of social networking addiction in participants

Intensity of social network addiction	*n* (%)
Natural use	7(1.9)
Mild addiction	57(15.8)
Moderate addiction	254(70.6)
Severe addiction	42(11.7)

**Table 3:** The correlation between social networking addiction and GPA in study samples

variables	GPA	
	*r*	*p*-Value
Salience	− 0.148^**^	0.005
Tolerance	− 0.133^*^	0.012
Mood modification	−0.171^**^	0.001
Relapse	−0.215^**^	0.000
Withdrawal	−0.164^**^	0.002
Conflict	−0.205^**^	<0.001
Total of social networking addiction	−0.210^**^	<0.001

^**^Correlation is significant at the 0.01 level (2-tailed)

^*^Correlation is significant at the 0.05 level (2-tailed)

Social networks play a crucial role in learning environments as a key communicational channel and a source of social support [11]. Many social networking websites, such as Edmodo, are specifically designed for learning [12]. Social networks have many advantages in learning as they provide wide access to information and information resources, reduce barriers to group interaction and telecommunications [13], support collaborative learning activities [14], encourage learners to learn more about self-learning [15], increase engagement and learner’s motivation [16], enhance engagement of learners with each other and their teachers [17] and support active and social learning [15]. In general, the emergence of new technologies such as internet and social networks, in addition to providing opportunities in facilitating and improving the quality of global communications, has created some threats [18]. When the use of social networks is managed poorly, they can have negative consequences at the individual and social levels. Social networking addiction is one of the consequences that many social network users may experience [19]. Thus, the extensive use of social networks is a new form of soft addiction [20].

There are many different theories about the addiction to internet and social networks. The most important theories include dynamic psychology theory, social control theory, behavioral explanation, biomedical explanation, and cognitive explanation. According to dynamic psychology theory, the roots of social networking addiction are in the psychological shocks or emotional deficiencies in childhood, personality traits, and psychosocial status. According to the social control theory, since addiction varies in terms of age, sex, economic status, and nationality, certain types of addiction are more likely to be found in certain groups of society than in other groups [21]. The theory of behavioral explanation believes that, a person uses social networks for rewards such as escaping reality and entertainment. Based on the biomedical explanation theory, the presence of some chromosomes or hormones, or the lack of certain chemicals that regulate brain activity, are effective in addiction [22, 23]. According to the cognitive explanation theory, social networking addiction is due to faulty cognition, and people tend to use social networks to escape from internal and external problems [24]. In general, addiction to social networking is classified as a form of cyber-relationship addiction [25].

Social networking addiction refers to mental concern over the use of social networks and the allocation of time to these networks in such way that, it affects other social activities of individuals such as occupational and professional activities, interpersonal relationships and health [19] leading to disruption of their life [20].

Social networking has a negative impact on physical and psychological health and causes behavioral disorders [26], depression [27, 28], anxiety and mania [28]. In this regard, results of a study on German students (2017) showed a positive relationship between addiction to facebook, with narcissism character, depression, anxiety and stress [29]. It is believed that addiction to social networking is higher in people with anxiety, stress, depression and low self-esteem [4]. Grifith (2005) suggests that addictive behavior is a behavior that has certain characteristics such as salience, mood modification, tolerance, withdrawal symptoms, conflict, and relapse [30]. Addictive behavior refers to repeated habits that increase the risk of a disease or social problems in a person. Over the past decade, addictive behaviors, such as overuse of internet or social networks, have become a part of everyday life of students. Social networking addiction includes the characteristics such as ignoring the real problems of life, neglecting oneself, mood swing, concealing addictive behaviors, and having mental concerns [4].

In this regard, signs and symptoms of addiction to social networking can include experiencing disturbances in day-to-day work and activities, spending more than one hour a day on social networks, being curios to see the old friends’ profiles, ignoring work and daily activities due to the use of social networks, and feeling anxious and stressed due to the lack of access to social networks [31].

Evidence suggests that many factors are associated with addiction to internet and social networks. Among these factors are online shopping, dating, gaming and entertainment, using mobile phones for access to internet, searching for pornographic images, user personality trails, and low self-esteem [19, 30, 32–34].

Students are one of the most important users of the virtual world and social networks. The overuse of social networks has positive and negative academic, social, and health consequences for the students [35]. Reduced academic performance is one of the most important consequences of social networking overuse for students. The results of a study on medical students showed that students who used social networks and internet more than average had a poor academic achievement and low level of concentration in the classroom [36]. The results of another study on Qatari students showed that Grade Point Average (GPA) was lower among students who were addicted to social networking compared to other students [37]. The results of a study in India showed that internet and social networking addiction had a negative effect on academic performance and mental health of students [38]. The results of a Korean study revealed a negative correlation between the use of internet for non-academic purposes and academic performance of students [39]. Findings of a study in Iran (2018) also showed a significant correlation between addiction to the internet and educational burnout [40].

Thus, considering the key role of students in promoting the quality of physical and mental health of society, and also due to the lack of knowledge on the type of relationship between social networking addiction and academic performance of the students of medical sciences in Kermanshah University of Medical Sciences (KUMS), the present study was designed and implemented. The purpose of this study was to investigate the extent of social networking addiction among the students of medical sciences and its relationship with academic performance of the students.

Thus, we sought to examine the following hypothesis in this study:

1) There is significant relationship between the mean of social networking addiction and students gender.

2) Social networking addiction have a negative and significant correlation with academic performance.

## Methods

### Design

This descriptive-analytical and cross-sectional study was conducted between June and August 2018.

### Sample and sampling method

The research population consisted of all students who were studying at KUMS in the second semester of 2017–2018 academic years. The criteria for entering the study included; studying at the second semester of 2017–2018 academic year, studying at the second semester or above, willing to participation in the study, and completing the questionnaires fully. Stratified random sampling was performed. To calculate the sample size, the result of Masterz’s study (2015) was used [41], according to which, addiction to Facebook, Twitter and YouTube social networks was 14.2, 33.3, and 47.2, respectively. If we assume that, the prevalence of social networking addiction is about 33.3%, then the sample size will be 340 individuals considering 10% drop out of the samples. Thus, in the present study, in order to increase the stability and accuracy of the results, 360 participants using random sampling method were entered into the study.

### Instruments

The study tools included a personal information form and the Bergen Social Media Addiction Scale (BSMAS). The information form had 5 questions about gender, age, educational level, school of study, and Grade Point Average (GPA). BSMAS was designed by Andreassen et al. (2012) at the University of Bergen [42]. The reliability coefficient of this questionnaire has been confirmed by the Cronbach’s Alpha method (alpha = 0.8), [42] and its internal consistency has been calculated to be 0.88 [43]. The psychometric properties of the Persian version of the BSMAS using confirmatory factor analysis and Rasch models on 2676 students by quota sampling, have been reviewed and approved in Iran by reporting the indexes such as X^2^ = 86.52 (*P* < 0.001), CFI = 0.993, Average variance extracted = 0.51, and composite reliability = 0.86 [44]. In the present study, the reliability coefficient of the questionnaire for internal consistency was 0.88 using Cronbach’s Alpha method.

BSMAS consists of 18 questions and 6 items, in a way that, each item has 3 questions. The items include; salience [1–3], tolerance [4–6], mood modification [7–9], withdrawal [10–12], relapse [13–15] and conflict [16–18]. Salience refers to our thinking and behavior in using social networks. It means that, the addictive use of social networks is manifested in the form of individual’s dependency on social networks. Tolerance (craving) represents a gradual increase in the use of social networks to gain pleasure. Mood modification represents modifying and improving behavior or mood. In other words, this component suggests that some users use social networks to get rid of unpleasant feelings. Withdrawal is an unpleasant feeling that a person experiences when disconnected from social networks or discovers he or she is forbidden to use social network. Relapse is a failed attempt of a person to control his/her social networking usage. Conflict represents issues that cause tensions in relationships with others, workplace, education, etc. [42, 43].

The questions in this scale are in 5-point Likert scale, including very rarely [1], rarely [2], sometimes [3], often [4] and very often [5], which are scored from 1 to 5, respectively. The minimum score in the Social Networking Scale is 18 and the maximum score in 90. In our study, the average response time to the questionnaire was about 20 min. The questionnaires were distributed in faculties at the end of the classes. The sampling lasted for one month.

In this study, the samples were categorized in one of the following categories according to the score they obtained from the questionnaire: Normal use of social networks (0–19), mild social networking addiction [20–35, 43, 45–47], moderate social networking addiction (40–69) and severe social networking addiction (70–90), [48]. GPA was used to assess the academic performance of students.

### Data collection

At first, the study permission was obtained from the KUMS’s Research Deputy. Then, the researcher attended the Department of Education at the faculties of KUMS, including the faculties of Medicine, Para medicine, Dentistry, Pharmacy, Nursing and Midwifery and Health, and received a list of students from each faculty. The list was numbered and then, based on random number table method, samples were selected. The researcher referred to the students based on their classroom schedule and, if they were interested in participating in the study, invited them to enter the study. If any student did not want to participate in the study, he/she was replaced by the next or pervious person in the list. The objectives of the study were explained to all samples and then the questionnaires were given to them to be complete. The questionnaires were collected after the completion.

### Data analysis

Data were analyzed by 18th version of the Statistical Package for Social Sciences (SPSS Inc., Chicago, IL, USA) and two levels of descriptive and inferential statistics. The data normality was first evaluated using Kolmogorov-Smirnov test, which indicated an abnormal distribution of variables of social networking addiction and GPA. Spearman’s correlation coefficient was used to examine the correlation between the social networking and GPA. To compare the social networking addiction scores in terms of nominal qualitative variables (such as sex), the Mann-Whitney U test was used, and in terms of ordinal qualitative variables (such as education level and school) and quantitative variables (such as age and group), Kruskal-Wallis H test was used. *p*-value of less than 0.05 was considered as significant level.

### Ethical consideration

The University’s Ethics Committee approved the study with the code: IR.KUMS.REC.1397.077. The goals of study were explained to the samples and written informed consent was obtained from all of them. Concerning the confidentiality of personal information and responses, reassurance was given to the participants.

### Findings

Of the 360 ​​students participating in the study, 199 students (55.3%) were female and the rest were male. The mean age of the participants was 25.48 ± 3.39 years and they were mainly at the age range of between 21 and 30 years old. Also, 46.7% of the students (*n*=168) were undergraduate and most of them were studying at the faculty of dentistry (*n*=101, 28.1%), (Table 1).

The mean social networking addiction was 50.83 ± 13.00 out of 90, which was at moderate level. Most of the students had moderate addiction (254 students and 70.6%), (Table 2). The addiction to social networking in the male students was significantly higher than female students (*p* ≤ 0.01), (with the mean and standard deviation of 52.65 ± 11.50 and 49.35 ± 13.96, respectively). In term of age, the highest and lowest levels of social networking addiction were related to age groups of less than 20 years old and 31 to 40 years old (with the mean of 53.78 ± 14.95 and 50.57 ± 11.45, respectively), which showed no statistically significant difference. Undergraduate and PhD students had the highest and lowest level of addiction, respectively, and did not have statistically significant difference (with the mean and standard deviation of 52.8 ± 12.70 and 48.03 ± 13.95, respectively). In terms of school, the highest and lowest levels of addiction were related to the students of Para medicine and nursing and midwifery schools, respectively (with a mean and standard deviation of 53.49 ± 12.53 and 48.08 ± 13.67, respectively), and this difference was not statistically significant. (Table 1). There was a negative and significant correlation between social networking addiction and academic performance (*p* ≤ 0.01, r = − 0.210) of the students. Also, there was a negative and significant correlation between all the subscales of social networking addiction and GPA (Table 3).

## Discussion

In our study, the rate of addiction to social networking was moderate. In this regard, the prevalence of social networking addiction among students in Singapore and India was reported to be 29.5 and 36.9% respectively [26, 28]. The results of a meta-analysis study (2018) on ​​internet addiction showed that, the prevalence of internet addiction among medical students was 30.1% worldwide [49]. Results of a meta-analysis study (2017) suggest that, the prevalence of internet addiction in Iran is moderate [50]. Social networking addiction increases the incidence of disorders such as depression, stress and anxiety [28, 29]. If students fail to manage the time they spend on social networks and the reasons for doing that, they will be seriously harmed at individual and social levels. Accordingly, the result of a study showed that the overuse of social networks affects the social life of individuals [51]. Hawi and Samaha (2016) argued that, the higher the social networking addiction of students, the lower their self-esteem is [52]. The use of social networks has become an integral part of the lives of many students, because they introduce them to a world of different possibilities, especially in their field of study. However, these networks are like double-edged knives. If students do not manage the use of these networks, they will be addicted to them, and will have to face different consequences, especially in relation to their education.

Based on our findings, the first hypothesis of the study was confirmed and a statistically significant relationship was found between social networking addiction and students’ gender. In this regard, we found that the mean of social networking addiction in male students was significantly higher than female students. This part of our findings is consistent with the findings of other studies [26, 33, 34, 36, 45]. In studies conducted on students of medical sciences in Iran, internet addiction in male students was higher than female students [33, 53, 54]. Findings of a study in Turkey (2016) suggested that addiction in Tweeter social network among male students was higher than female students [55]. But the results of a study on Polish students showed that, female students were using Facebook more than male students [46], Andreassen et al. (2017) showed that being female is one of the factors that has a statistically significant relationship with social networking addiction [43]. According to the social control theory, since addiction varies in terms of demographic variables such as sex, certain types of addiction are more likely to be found in certain groups of society than in other groups [21]. In this regard, evidence suggests that in general, 68% of women and 62% of men use social networks, and on average, women spend 46 min and men spend 31 min on social networking [52].

Based on the findings, the second hypothesis of the research was confirmed and a negative and significant correlation was found between social networking addiction and students’ academic performance. This finding means that, an increase in the excessive use of social networks decreases the academic performance. Based on the theory of behavioral explanation, a person enters social networks for rewards such as escaping reality and entertainment [21]. Excessive use of these networks can cause addiction in the user. Our results are consistent with the findings of Ahmadi and Zeinali (2018), Kumar et al. (2018), and Kim et al. (2018) studies [38, 39, 56]. In this regard, Ahmadi and Zeinali (2018) in a study showed that social networking addiction has a negative impact on academic achievement by creating academic procrastination, reducing sleep quality and increasing academic stress [56]. However, Junco et al. (2011) believed that some social networks such as Twitter can be used as a learning tool by students and professors. Also, these networks can increase academic engagement in students and professors [57]. But the point about the use of social networks as an educational tool is that, overuse of social networks reduces the level of academic engagement and students’ grades. Therefore, when using social networks, special attention should be paid to the time management. In fact, improving students’ academic performance depends on the lesser use of social networks [58]. Evidence suggests that excessive use of social media such as Facebook is associated with a significant level of stress and this stress, negatively affects the student’s academic performance [59]. Uncontrolled use of social media reduces the study time, which has a negative effect on the academic performance of students. Also, since people who spend many hours around the clock using social media do not have enough rest and suffer from fatigue and sleep disruption, these can have a negative impact on their concentration and learning [60]. Reducing the quality of sleep, negatively affects the students’ concentration and academic quality. Additionally, reducing the duration of sleep may interfere with the secretion of serotonin and melatonin, and this increases the level of stress and anxiety of students. As a result, these hormonal changes reduce brain function and cognitive abilities [56]. In line with these studies, evidence indicates a positive and significant correlation between inappropriate and problematic use of technology and educational problems [61, 62]. In fact, the over-use of social networks will result in failure in education and social relationships, and also leads to ineffective time management. Social media is not self-destructive and harmful on its own, but rather it is the way of using it that leads to positive and negative consequences. The proper use of social media requires a culture and awareness of how they should be used correctly. In this regard, the results of a study indicated that universities that use this technology can motivate students in the specialized field to help them be effective and positive. Increasing students’ motivation can lead to progress in different areas, especially education [63]. Despite this issue, some university professors and lecturers still oppose the use of social networks by students [64]. In our opinion, the increasing expansion of social networks has provided opportunities and unique conditions for the growth and improvement of students’ academic status, but they should be used sensitively and managed properly, because due to the attractiveness of various social networks, it is possible to get addicted to them.

### Limitations

Our study had several limitations. Due to the cross-sectional nature of this study, it was not possible to explain the causal relationships between the variables of social networking addiction and academic performance of students. In the current study, the data were collected by self-reporting method that could have affected the accuracy of the results. However, the researcher tried to solve this limitation by reassuring the participants that their responses would remain confidential.

### Practical implications

Since students, who have a high level of anxiety, stress, and depression and a low level of self-esteem, are more at risk of social networking addiction, designing and implementing counseling programs to promote mental health is recommended for them. Additionally, Cognitive Behavioral Therapy (CBT) is suggested to reduce social networks dependency. CBT is one of the most effective therapies for reducing social networks dependency. Based on the CBT method, thoughts are the determinant of emotion, therefore, by controlling negative thoughts and managing behavior, we can reduce the dependence on social networks.

## Conclusions

The level of social networking addiction of the students was moderate, and male students had a higher level of addiction to social networking than female students. A significant and negative relationship was found between the social networking addiction and GPA. Considering the negative effects of social networking on students’ academic performance, the issue of addiction to social networking should be comprehensively reviewed and considered. Also, appropriate planning should be made to prevent addiction to social networking, control its use, and increase the opportunities and reduce the threats of this tool. In this regard, allocating some of the research priorities to the positive and negative applications of social media at individual, social and academic levels can be beneficial. Given the importance of addiction to social networking and its potentially destructive impact on students’ academic performance, similar studies are recommended in other universities and in different fields to obtain a more conclusive result. In this regard, the use of mix methods can help to better understand the phenomenon of addiction to social networking and its relationship with the academic performance of students.

## Abbreviations

BSMAS: Bergen Social Media Addiction Scale
GPA: Grade Point Average
KUMS: Kermanshah University of Medical Sciences
SPSS: Statistical Package for the Social Sciences
